# The Automatic but Flexible and Content-Dependent Nature of Syntax

**DOI:** 10.3389/fnhum.2021.651158

**Published:** 2021-06-11

**Authors:** Laura Jiménez-Ortega, Esperanza Badaya, Pilar Casado, Sabela Fondevila, David Hernández-Gutiérrez, Francisco Muñoz, José Sánchez-García, Manuel Martín-Loeches

**Affiliations:** ^1^Cognitive Neuroscience Section, UCM-ISCIII Center for Human Evolution and Behavior, Madrid, Spain; ^2^Department of Psychobiology & Behavioral Sciences Methods, Complutense University of Madrid, Madrid, Spain; ^3^School of Philosophy, Psychology and Language Sciences, The University of Edinburgh, Edinburgh, United Kingdom

**Keywords:** unconscious processing, left anterior negativity, automaticity of syntax, unconscious emotion perception, syntax flexibility

## Abstract

Syntactic processing has often been considered an utmost example of unconscious automatic processing. In this line, it has been demonstrated that masked words containing syntactic anomalies are processed by our brain triggering event related potential (ERP) components similar to the ones triggered by conscious syntactic anomalies, thus supporting the automatic nature of the syntactic processing. Conversely, recent evidence also points out that regardless of the level of awareness, emotional information and other relevant extralinguistic information modulate conscious syntactic processing too. These results are also in line with suggestions that, under certain circumstances, syntactic processing could also be flexible and context-dependent. However, the study of the concomitant automatic but flexible conception of syntactic parsing is very scarce. Hence, to this aim, we examined whether and how masked emotional words (positive, negative, and neutral masked adjectives) containing morphosyntactic anomalies (half of the cases) affect linguistic comprehension of an ongoing unmasked sentence that also can contain a number agreement anomaly between the noun and the verb. ERP components were observed to emotional information (EPN), masked anomalies (LAN and a weak P600), and unmasked ones (LAN/N400 and P600). Furthermore, interactions in the processing of conscious and unconscious morphosyntactic anomalies and between unconscious emotional information and conscious anomalies were detected. The findings support, on the one hand, the automatic nature of syntax, given that syntactic components LAN and P600 were observed to unconscious anomalies. On the other hand, the flexible, permeable, and context-dependent nature of the syntactic processing is also supported, since unconscious information modulated conscious syntactic components. This double nature of syntactic processing is in line with theories of automaticity, suggesting that even unconscious/automatic, syntactic processing is flexible, adaptable, and context-dependent.

## Introduction

Traditionally, syntactic processing has been considered a paramount example of unconscious automatic processing (Fodor, 1983; Hauser et al., 2002), an idea supported by empirical evidence (Hasting and Kotz, 2008; Batterink and Neville, 2013; Jiménez-Ortega et al., 2014; Lucchese et al., 2017). Current refined theories of automaticity suggest that unconscious/automatic processing is flexible, adaptable, context-dependent, and able to influence a variety of cognitive and executive functions (For reviews see:Kiefer, 2012; Ansorge et al., 2014). However, little is known on whether syntax would display these characteristics. In the present study, we aim to investigate whether syntax might have an automatic but flexible and content-dependent nature.

Recent views of brain function and neuroanatomy have described highly overlapping networks between emotion and cognition (Pessoa, 2012, 2016). Emotional information, regardless of the level of awareness, has an enormous adaptive value, probably interacting with many cognitive domains, such as planning, attention, memory, decision-making, or language (Ashby et al., 1999; Mitchell and Phillips, 2007; Pessoa, 2008; Vissers et al., 2010; Jiménez-Ortega et al., 2012; Martin-Loeches et al., 2012). Evidence suggests that a large part of emotional information is processed automatically, without awareness. Following that unconsciously processed stimuli impact cognitive processes at several levels, such as language comprehension and decision-making (for reviews see:Dehaene et al., 2006; Van den Bussche et al., 2009; Kiefer et al., 2011; Smith et al., 2015; Perlovsky and Schoeller, 2019), evidence has shown that unconscious emotional information can as well affect processes such as decision-making, semantics, and syntax, and trigger long-lasting cerebral processes (Naccache et al., 2005; Gibbons, 2009; Yang et al., 2017). This supports an extensive and rich interweaving between emotion and cognition, even at the unconscious level.

Recent evidence challenges the traditional view that syntax is an encapsulated process (Fodor, 1983; Hauser et al., 2002). For example, syntactical processing of sentences has been recently reported to be affected by emotions with and without awareness (Vissers et al., 2010; Martin-Loeches et al., 2012; Hinojosa et al., 2014; Verhees et al., 2015; Jiménez-Ortega et al., 2017, 2020). Likewise, the mere social presence of a confederate has been shown to elicit syntactic modulations (Hinchcliffe et al., 2020). Overall, the influence of extralinguistic cues such as emotions and social presence on syntax and regardless of the level of awareness seem to be supported.

Modulations of event related potential (ERP) components allow us to investigate the unconscious emotional effect on syntax, due to their high temporal resolution. Anterior negativities appear between 300 and 500 ms after stimulus onset over frontal electrodes to grammatical incongruences, such as morphosyntactic anomalies (e.g., Rothermich et al., 2010; Steinhauer and Drury, 2012; Bohn et al., 2013; Magne et al., 2016). They are typically left-sided, so often it is denominated LAN (left anterior negativity), though fronto-central distributions might also appear (Molinaro et al., 2015). These negativities seem to reflect highly automatic first-parsing processes, the detection of morphosyntactic mismatches, the difficulty of processing correct but rare grammatical structures (Friederici et al., 1999; Friederici and Kotz, 2003), and even some working memory operations (King and Kutas, 1995; Weckerly and Kutas, 1999; Martin-Loeches et al., 2005; Makuuchi et al., 2009). As reviewed in Friederici (2017), the LAN is most probably originated in area BA44 of the left inferior frontal gyrus, which, together with the posterior superior temporal sulcus, underlies syntactic processing (Matchin et al., 2017; Schell et al., 2017).

A more general marker for structural processing, the later (500–1000 ms) centro-parietal positive component (P600), is said to reflect a later-stage syntactic reanalysis and reintegration (Osterhout et al., 1994; Hagoort et al., 2003; Molinaro et al., 2011). More recently, it has been suggested that it may also reflect integration processes of conscious and unconscious linguistic information (Jiménez-Ortega et al., 2014). The semantic P600, when found, has also been linked to the assessment of incoming information to update the mental model (Burkhardt, 2007). The P600 probably emerges in the angular gyrus and the posterior superior temporal gyrus (Friederici, 2017; Schell et al., 2017).

Particularly, the effects of emotional language on syntactic processing have been previously reported for both the LAN and the P600 (Vissers et al., 2007, 2010; Jiménez-Ortega et al., 2012; Martin-Loeches et al., 2012; Van Berkum et al., 2013; Fraga et al., 2017). Emotional effects on the LAN may indicate that the lexical–semantic information conveyed by emotional linguistic stimuli can impact syntactic processing at its early—and presumably automatic—stages (Hasting and Kotz, 2008; Batterink and Neville, 2013; Jiménez-Ortega et al., 2014; Lucchese et al., 2017). However, how emotional information affects syntax is unclear. While some studies observed a similar LAN component between positive/pleasant and negative/unpleasant information (Jiménez-Ortega et al., 2012; Espuny et al., 2018a; Padron et al., 2020), others observed differences at early components to syntactic anomalies between positive and negative information (Martin-Loeches et al., 2012; Jiménez-Ortega et al., 2017; Espuny et al., 2018b). This variability might depend on both the arousal and valence of the emotional material (Citron et al., 2013; Hinojosa et al., 2020). For example, moderately arousing pleasant and unpleasant adjectives containing syntactic anomalies with respect to an ongoing sentence have been found to trigger similar LAN components (Padron et al., 2020). In contrast, Espuny et al. (2018b), using also moderately arousing words, observed that negative valanced words triggered a larger LAN than positive and neutral valanced words. Nonetheless, in this case, the emotional words were presented preceding sentences containing gender and number anomalies instead within the sentence. Therefore, other factors, such as the moment and mode of the emotional information presentation, may also account for these dissimilarities.

Relevant to our aims, LAN modulations have also been observed to unconscious emotional information. Specifically, the unconscious processing of either emotional adjectives or facial expressions showed a distinct effect on the onset of the LAN and its topography (Jiménez-Ortega et al., 2017, 2020). Evidence also points out that masked syntactic anomalies trigger similar ERP components to those elicited by consciously perceived syntactic anomalies (Jiménez-Ortega et al., 2014). Since unconscious perception ensures automatic processing (Kiefer et al., 2012), the observed components to such unconscious anomalies support the automatic nature of syntax, as proposed by other authors using different approaches, such as attentional blink (Hasting and Kotz, 2008; Pulvermuller et al., 2008; Lucchese et al., 2017).

As mentioned above, it has been demonstrated that both conscious and unconscious emotions and social context can affect language comprehension and particularly syntactic processing (Jiménez-Ortega et al., 2012, 2017, 2020; Van Berkum et al., 2013; Verhees et al., 2015). Therefore, early syntactic parsing might be automatic but can be modulated by emotional and social cues. This is in line with theories of automaticity that suggest that even unconscious/automatic processing is flexible, adaptable, and context-dependent. Unconscious information can influence a variety of cognitive and executive functions, probably as a function of high-level executive control settings (For reviews see:Kiefer, 2012; Ansorge et al., 2014). Yet, there are few studies currently investigating this automatic but flexible conception of syntactic parsing. Although automatic processing can occur with or without awareness, a suitable way to investigate automatic processing is by presenting unaware masked stimuli (Ansorge et al., 2014). Hence, to investigate this automatic but flexible conception of syntax, we examined whether and how masked emotional words (positive, negative, and neutral masked adjectives) containing morphosyntactic anomalies (half of the cases) affect the linguistic comprehension of ongoing unmasked sentences that also contain, in half of the cases, a number disagreement anomaly between the noun and the verb. Following the canonical order in Spanish, the masked adjectives always appeared between the unmasked noun and verb.

If, as discussed above, early syntactic parsing is automatic, we expect to observe syntactic components following masked anomalies (number disagreement between a masked adjective and an unmasked noun). If syntax is also flexible and content-dependent, the emotional content of the masked adjectives might also modulate the syntactic components to conscious number anomalies (number disagreement between an unmasked verb and an unmasked noun). Furthermore, we also expect interactions between unmasked syntactic anomalies (number disagreement between a masked adjective and an unmasked noun) and masked ones (number disagreement between an unmasked verb and an unmasked noun). Since the presence of an unconscious anomaly might prime the subsequent conscious anomaly, we expect an amplitude reduction of conscious syntactic components.

## Methods

### Participants

Twenty-six Spanish-native speakers (18 female, 8 male) participated in the experiment. Their age ranged from 18 to 26 years old (mean: 20.5) All participants were right-handed (mean score: +82, range: +20 to +100) according to the Edinburgh Handedness Inventory (Oldfield, 1971). Participants had normal or corrected-to-normal vision, no hearing difficulties, and no previous history of neural or psychiatric disorders. Participants gave their informed consent before the experiment, following the Declaration of Helsinki, as approved by the ethics committee of the Center for Human Evolution and Behavior (UCM-ISCIII) and were reimbursed afterwards.

### Materials

Three hundred sixty sentences were created as experimental stimuli following the Spanish canonical order of determiner – noun – verb. Between the noun and the verb, a masked adjective was presented for 8 ms, preceded and followed by a hash mark also lasting 74 ms. Such method demonstrated its efficacy in previous studies (Van den Bussche et al., 2009; Kiefer et al., 2012; Jiménez-Ortega et al., 2014, 2017). To increase sentence structure variability, half of the sentences were presented in a short format (i.e., adding a complement after the verb), and the other half were in a long format (adding to components after the verb). All sentences were simple clauses (see Table 1 for examples).

**TABLE 1:** Example stimuli for the short and long sentences.

			**Determinant**	**Noun**	**Subliminal adjective valence** **Positive/Neutral/Negative**	**Mask**	**Supraliminal verb** **Correct/Incorrect**	**Complement**	**Complement**
**Subliminal adjective agreement**	**Correct**	**Supraliminal verb agreement**	La	radio	conocida	#######	emite	noticias	
					nacional				
					insensible				
			*The*	*radio*_[__sing.]_	*known*_[sing.]_	#######	*broadcasts*_[sing.]_	*news*	
					*national*_[sing.]_				
					*insensitive*_[sing.]_				
		**Supraliminal verb disagreement**	La	radio	conocida	#######	emiten	noticias	
					nacional				
					insensible				
			*The*	*radio*_[__sing.]_	*known*_[sing.]_	#######	*broadcasts*_[plural]_	*news*	
					*national*_[sing.]_				
					*insensitive*_[sing.]_				
		**Supraliminal verb agreement**	Los	abrigos	buenos	#######	calientan	el	cuerpo
					étnicos				
					raídos				
			*The*	*coats*_[plural]_	*good*_[plural]_	#######	*warm*_[plural]_	*the*	*body*
					*ethnic*_[plural]_				
					*ragged*_[plural]_				
		**Supraliminal verb disagreement**	Los	abrigos	buenos	#######	calienta	el	cuerpo
					étnicos				
					raídos				
			*The*	*coats*_[plural]_	*good*_[plural]_	#######	*warms*_[sing.]_	*the*	*body*
					*ethnic*_[plural]_				
					*ragged*_[plural]_				
	**Incorrect**	**Supraliminal verb agreement**	La	radio	conocidas	#######	emite	noticias	
					nacionales				
					insensibles				
			*The*	*radio*_[sing.]_	*known*_[plural]_	#######	*broadcasts*_[sing.]_	*news*	
					*national*_[plural]_				
					*insensitive*_[plural]_				
		**Supraliminal verb disagreement**	La	radio	conocidas	#######	emiten	noticias	
					nacionales				
					insensibles				
			*The*	*radio*_[sing.]_	*known*_[plural]_	#######	*broadcast*_[plural]_	*news*	
					*national*_[plural]_				
					*insensitive*_[plural]_				
		**Supraliminal verb agreement**	Los	abrigos	bueno	#######	calientan	el	cuerpo
					étnico				
					raído				
			*The*	*coats*_[plural]_	*good*_[sing.]_	#######	*warm*_[plural]_	*the*	*body*
					*ethnic*_[sing.]_				
					*ragged*_[sing.]_				
		**Supraliminal verb disagreement**	Los	abrigos	bueno	#######	calienta	el	cuerpo
					étnico				
					raído				
			*The*	*coats*_[plural]_	*good*_[sing.]_	#######	*warms*_[sing.]_	*the*	*body*
					*ethnic*_[sing.]_				
					*ragged*_[sing.]_				

The same 360 sentences were used for all experimental conditions. Therefore, each sentence had 12 different versions, according to the three main conditions: correctness of the masked adjective (correct, incorrect), correctness of the verb (correct, incorrect), and valence of the masked adjective (negative, positive, neutral) (see Table 1 for examples), yielding a total number of 4320 experimental sentences. Stimuli presentation was counterbalanced across participants as described below.

The combination of grammatical correctness (regarding number agreement) in the unmasked sentence (between the noun and the verb) and the masked adjective (between this and the unmasked noun) led to four possibilities: (1) all correct (e.g., La radio_sing_ *conocida*_sing_ ###### emite_sing_ noticias/The radio_sing_ *known*_sing_ ###### broadcasts_sing_ news), (2) masked adjective over unmasked noun disagreement (e.g., La radio_sing_ *conocidas*_plural_ ###### emite_sing_ noticias/The radio_sing_ *known*_plural_ ###### broadcasts_sing_ news), (3) unmasked noun over unmasked verb disagreement (e.g., La radio_sing_ *conocida*_sing_ ###### emiten_plural_ noticias/The radio_sing_ *known*_sing_ ###### broadcast_plural_ news), and (4) all incorrect (e.g., La radio_sing_ *conocidas*_plural_ ###### emiten_plural_ noticias/The radio_sing_ *known*_plural_ ###### broadcast_plural_ news), that is, both masked adjective over unmasked noun and unmasked noun over unmasked verb disagreements. These conditions will be referred as follows, respectively: (1) Masked Correct – Unmasked Correct (C_NA_-C_NV_-C_AV_), (2) Masked Incorrect – Unmasked Correct (I_NA_-C_NV_-I_AV_), (3) Masked Correct – Unmasked Incorrect (C_NA_-I_NV_-I_AV_), and (4) Masked Incorrect – Unmasked Incorrect (I_NA_-I_NV_-C_AV_) (Table 2), where _NA_ refers to the noun-masked adjective number agreement, _NV_ refers to the noun–verb agreement, and _Av_ refers to the masked adjective–verb agreement.

**TABLE 2:** Experimental conditions arising for Masked Correctness by Unmasked Correctness factors regardless of Emotion factor.

		***Noun-verb (NV) (target task)***	
		**Correct (C)**	**Incorrect (I)**
***Noun-masked adjective (NA)***	**Correct (C)**	(1) C_NA_-C_NV_-C_AV_	(3) C_NA_-I_NV_-I_AV_
	**Incorrect (I)**	(2) I_NA_-C_NV_-I_AV_	(4) I_NA_-I_NV_-C_AV_

*Where _NA_ refers to the noun-masked adjective number agreement, _NV_ to the noun-verb agreement and _Av_ to the masked adjective-verb agreement.*

Valance (V) and arousal values were obtained from Stadthagen-Gonzalez et al. (2017). Five hundred twelve participants rated 14,031 Spanish words on a nine-point scale for valence [1 = infeliz (“unhappy”), 9 = feliz (“happy”)] and arousal [1 = tranquilo(a) (“quiet”), 9 = excitado (“excited”)]. Three versions of each of the above sentences were created to have them presented with a positive (V > 6.5, *M* = 7.3, *SD* = 0.45), neutral (6.45 > V > 4.9, *M* = 5.59, *SD* = 0.37), or negative (4.7 > V, *M* = 3.53, *SD* = 0.61) masked adjective. All the masked adjectives were matched for arousal (*Mpos* = 5.36, *SD* = 0.87; *Mneu* = 5.38, *SD* = 0.61; *Mneg* = 5.37, *SD* = 0.79) (Table 2). Therefore, the 4 conditions above turn into 12 conditions, as a function of the valence of the masked adjective: C_NA_-C_NV_-C_AV_-Pos, I_NA_-C_NV_-I_AV_-Pos, C_NA_-I_NV_-I_AV_-Pos, I_NA_-I_NV_-C_AV_-Pos, C_NA_-C_NV_-C_AV_-Neg, I_NA_-C_NV_-I_AV_ -Neg, I_NA_-I_NV_-C_AV_-Neg, I_NA_-I_NV_-C_AV_-Neg, C_NA_-C_NV_-C_AV_-Neu, I_NA_-C_NV_-I_AV_ -Neu, I_NA_-I_NV_-C_AV_-Neu, I_NA_-I_NV_-C_AV_-Neu.

Semantic acceptability between the masked adjective and the unmasked sentence was carefully controlled. Acceptability for each condition was the number of results in a Google search within quotation marks for “noun adjective” (e.g., “*detective privado*” –English: “*private detective*”-). The average acceptability of noun–adjective pairs, frequency, valence, and activation of the adjectives can be found at Table 3. No significant differences were found for any of the adjectives characteristics (all Fs < 1.44, *p* > 0.24) with the exception of valance [*F(2,718) = 4733.1, p* < 0.*001, η_p_^2^ = 0.929, θ = 0.1*], as intended. Although unmasked verbs also contained number anomalies with respect to the noun, their linguistically relevant variables were not controlled, since each unmasked noun–verb combination was presented in 12 conditions the same number of times.

**TABLE 3:** Means (SDs) for linguistically relevant variables in masked adjectives.

	**Valence**	**Arousal**	**Length**	**Frequency**	Acceptability*	**Participles %**
**Positive**	7.3 (0.45)	5.27 (0.87)	7.4 (1.5)	1248.23 (2056.5)	22439.87 (75896.63)	37,6
**Neutral**	5.59 (0.37)	5.38 (0.61)	7.5 (1.5)	1587.5 (4815.9)	26697.12 (26697.12)	30.6
**Negative**	3.45 (0.61)	5.37 (0.79)	7.4 (1.7)	817.3 (1688.9)	17880.81 (26697.12)	37.4

*Acceptability of masked adjective over unmasked noun combination, as measured by the number of hits appearing in a Google search.

Experimental sentences were distributed in 12 presentation sets so that each sentence was presented only in one condition per participant. Therefore, each presentation set contained one version of the 360 sentences, equally distributing the 12 conditions. Additional 360 sentences of different length and structure were added as filler stimuli, previously used in Jiménez-Ortega et al. (2017, 2020). Each filler could be correct or incorrect and contained strings of hash marks inserted at different places of the sentence, except between the noun and the verb, in order to increase variability relative to the experimental sentences. They were presented during 82 ms in order to generate a similar conscious perception to the one produced by the presentation of the forward hash mask, the masked adjective, and the backward hash mask in the experimental sentences.

### Procedure

Participants were told that the aim of the experiment was to investigate how people process grammatical errors. Their task was to indicate whether the sentence was grammatically correct or not by pressing one of two buttons with their index fingers. The assignment of correct button was counterbalanced. Participants performed a practice session with 20 sentences that were not presented in the experiment to check if they had understood the task. They were also instructed to avoid blinking and eye movements during the presentation of the sentences.

**FIGURE 1:**
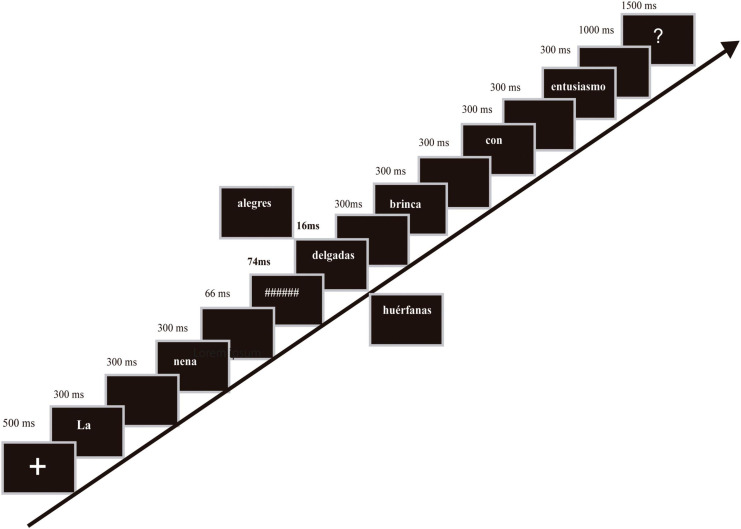
Experimental procedure. Sentences contained masked emotional adjectives (positive, negative, or neutral adjectives) that could be either correct or incorrect (number disagreement relative to unmasked nouns). In addition, the ongoing unmasked sentence could also be correct or incorrect (noun–verb number disagreement). In the example, the masked adjective was incorrect (examples of positive, negative, and neutral adjectives appear), while unmasked verb was correct. The literal translation into English of the example is: *The babe [thin/happy/orphan (all plural)] jumps with enthusiasm*.

Stimuli were presented white-on-black using the Presentation software on a 240Hz-HP-LCD screen, centered in the monitor screen, with visual angles around 0.8^*o*^ in height and 0.8^*o*^ to 14^*o*^ in width, at a viewing distance of 65 cm. As illustrated in Figure 1, each trial began with a fixation cross for 500 ms, after which the sentence was displayed word-by-word. Each word was displayed for 300 ms, except for the hash mask and masked adjective, which were presented for 74 and 16 ms, respectively. A blank screen was displayed after each word for 300 ms including the masked adjective, followed by a question mark after the last word that signaled participants to answer. Participants were given 1000 ms to respond. After that, the next trial began after 1000 ms. There was a pause every 180 trials to avoid fatigue. The experimental session lasted around 60 min, plus electrode preparation.

Once the experimental session finished, participants performed a visibility test to check whether participants were aware of the masked adjective. Following the procedure by Jiménez-Ortega et al. (2014, 2017), participants were presented with a subset (40 trials) of the experimental stimuli and fillers and had to report if they saw anything after the presentation of the hash mask. According to the visibility test, none of the participants was aware of the masked adjective presentation nor did they differentiate between fillers and experimental sentences. Finally, participants filled in the Edinburgh handedness questionnaire.

### EEG Recording and Data Processing and Analysis

EEG data was recorded from 59 scalp, 4 EOG, and 2 mastoid electrodes using the standard 10/20 system at a sample rate of 250 Hz and a bandpass of 0.01–100 Hz. Scalp electrodes were referenced online to the left mastoid electrode – M1. They were later re-referenced offline to the average mastoid and re-filtered with a bandpass filter of 0.01–40 Hz. The recorded activity of bipolar vertical and horizontal electrooculograms (EOG) monitored eye-related activity such as eye movements and blinks. Electrode impedance kept below 5 KΩ.

The EEG data was analyzed with Brain Vision Analyzer^®^ software. The continuous EEG was segmented into epochs of 1400 ms for each trial, including a 200 ms baseline relative to the presentation of the masked adjective. Ocular correction was performed through Independent Component Analysis (ICA; Jung and Lee, 2007) as implemented in the software. Remaining artifacts were semi-automatically rejected by eliminating epochs exceeding +/−100 μV in any of the channels. Epochs that contained incorrect responses were removed from the data analysis. The average of artifact-free trials correctly answered per condition was 23.6 with no differences across conditions according to a Masked Correctness (2) × Unmasked Correctness (2) × Emotion (3) repeated ANOVA (all *Fs* < 1.079, *ps* > 0.3).

### Data Analysis

Factorial cluster analyses seem to successfully and objectively estimate time-windows for ERP components. Time-windows analyses might facilitate comparison with previous results, simplifying *post hoc* analyses. Therefore, given their advantages, analysis was done performing both techniques (For further details see: Groppe et al., 2011a, b; Brusini et al., 2016, 2017; Fields and Kuperberg, 2019). As a result, time-windows analyses were guided by cluster analyses results and further confirmed by visual inspections.

Cluster-based permutation analyses were calculated by using the Factorial Mass Univariate Toolbox (Fields and Kuperberg, 2019) on Matlab^®^, which builds upon and extends the Mass Univariate Toolbox developed by Groppe et al. (2011b). It allows complex factorial designs and shows good statistical power when *a priori* time segments are used (Fields and Kuperberg, 2019). Guided by Brusini et al. (2016); Jiménez-Ortega et al. (2020)’s procedures, two-time segments were considered for analyses, 0–600 ms and 600–1200 ms, to respectively capture the early and late components typically elicited by syntactic anomalies. Therefore, two factorial cluster-based permutation analyses were calculated, each with 10,000 iterations and an alpha level of 0.05 involving the factors Unmasked Correctness (correct, incorrect), Masked Correctness (correct, incorrect), and Emotion (positive, negative, and neutral).

**FIGURE 2:**
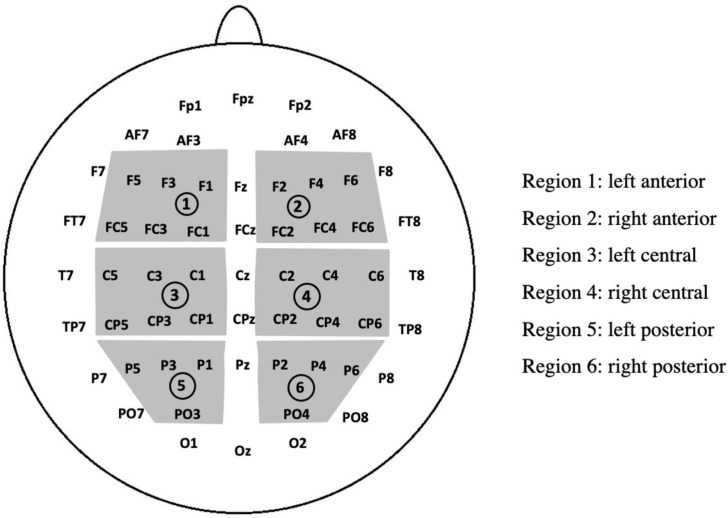
Scheme of the ROIs used for statistical analyses.

For the time-windows analyses, related statistical analyses of variance (ANOVAs) were performed with the SPSS 22^®^ software. Three brain regions of interest (ROIs) were used: anterior, central, and posterior regions (Figure 2). Thus, the ANOVA included five factors: ROI (anterior, central, posterior), Hemisphere (right, left), Unmasked Correctness (correct, incorrect), Masked Correctness (correct, incorrect), and Emotion (positive, negative, neutral). Similarly, behavioral data were analyzed using an Unmasked Correctness (2) × Masked Correctness (2) × Emotion (3) repeated-measures ANOVA.

Violations of the sphericity assumption were corrected if found by the Greenhouse–Heisser correction, and Bonferroni corrections were used for multiple comparisons. Guided by cluster analyses and confirmed by visual inspections, the following windows were analyzed: 150–230 ms, 470–550 ms, 860–960 ms, and 1050–1150 ms.

## Results

### Behavioral Data

Participants answered correctly 95.58% of the experimental trials. Although error rates were slightly larger for unmasked incorrect trials than for correct ones (1.7 vs. 0.9 respectively on average), the ANOVA analyses revealed no significant effects for error rates (all Fs < 3.4, *p* > 0.07). Likewise, no significant effects were observed for reaction times (all Fs < 1.8, *p* > 0.17).

### Cluster Analyses

The early time segment (0–600 ms) yielded a main effect of Unmasked Correctness at around 350 ms involving up to 30 electrodes mostly localized on anterocentral regions. A main effect of Emotion was observed at around 360 ms, which seems stronger at frontal left hemisphere, involving up to 30 electrodes (330–430 ms window). Similarly, a main emotional effect was observed at around 490 ms but involving up to 40 electrodes, which appears stronger at anterior electrodes, but also at right parietal ones (470–550 ms window). Finally, a Masked X Unmasked correctness significant interaction was found at 180 ms involving up to 40 electrodes (150–230 ms window) (Supplementary Figure 1).

Cluster-based permutation analyses at the later time segment (600–1200 ms) yielded revealed a main effect of Unmasked Correctness approximately at around 900 and also at around 1100 ms. Both clusters are broadly frontal-to-parietal distributed, involving up to 40 electrodes (Supplementary Figure 2).

### Time-Window Analyses

#### Time Window 150–230 ms

**FIGURE 3:**
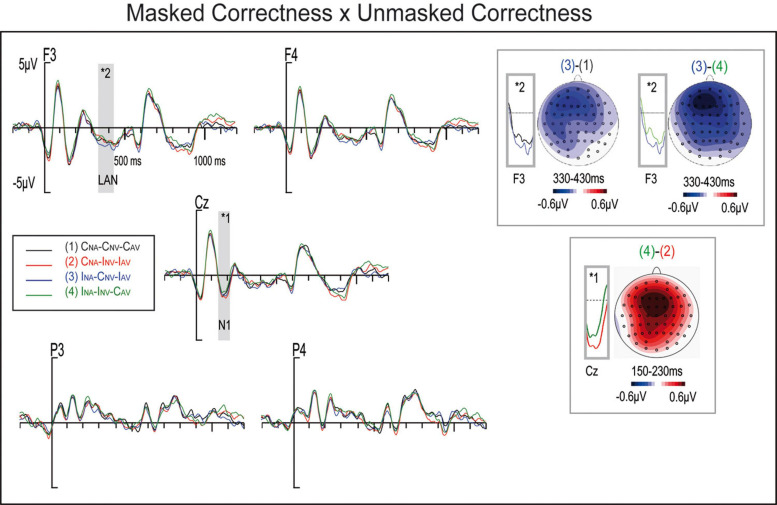
ERP waveforms at selected electrodes for conditions: (1) Masked Correct – Unmasked Correct (C_NA_-C_NV_-C_AV_), (2) Masked Incorrect – Unmasked Correct (I_NA_-C_NV_-I_AV_), (3) Masked Incorrect – Unmasked Correct (I_NA_-C_NV_-I_AV_), and (4) Masked Incorrect – Unmasked Incorrect (I_NA_-I_NV_-C_AV_). Difference maps of the N2 at 150–230 ms (I_NA_-I_NV_-C_AV_ minus C_NA_-I_NV_-I_AV_) and LAN at 330–430 ms time windows (I_NA_-C_NV_-I_AV_ minus C_NA_-C_NV_-C_AV_ and I_NA_-C_NV_-I_AV_ minus I_NA_-I_NV_-C_AV_ from left to right).

A visual inspection of the waveforms (Figure 3), supported by the cluster analyses, showed ERP modulations at the interval 150–230 ms. An ANOVA analysis in this interval found main Masked Correctness effects *(F(1,25) = 6.75, p = 0.015, η_p_^2^ = 0.21, θ = 0.705)*, where masked incorrect trials triggered smaller amplitudes than masked correct ones (Figure 3). Additionally, in line with cluster analyses, a significant Masked by Unmasked Correctness interaction *(F(1,25) = 10.85, p = 0.003, η_p_^2^ = 0.303, θ = 0.886)* was found, where the amplitude was significantly smaller for I_NA_-I_NV_-C_AV_ than for I_NA_-I_NV_-C_AV_ *(Δ = −0.530, p = 0.002)* (Figure 3). This difference might account for the above reported Masked main effect of correctness since Masked Incorrect sentences collapses I_NA_-I_NV_-C_AV_ and I_NA_-C_NV_-I_AV_ conditions, while Masked Correct sentences agglutinates C_NA_-C_NV_-C_AV_ and I_NA_-I_NV_-C_AV_. Additionally, C_NA_-C_NV_-C_AV_, I_NA_-C_NV_-I_AV_, and I_NA_-I_NV_-C_AV_ did not differ between them, as can be observed in Figure 3 as well as confirmed by non-significant post-hoc comparisons. All other main effects of factors and their interactions did not reach significance (all *Fs < 2.1, ps > 0.13*).

#### Time Window 330–430 ms

**FIGURE 4:**
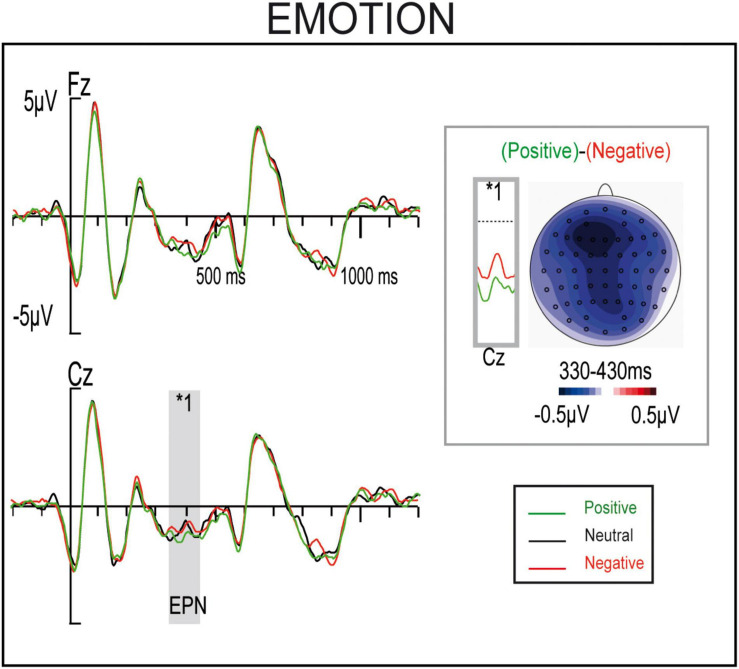
ERP waveforms and maps for sentences containing either positive, neutral, or negative masked adjectives.

In line with cluster analyses, the ANOVA analyses at 330–430 ms yielded significant effects for Region × Unmasked Correctness *(F(2,50) = 4.16, p = 0.046, η_p_^2^ = 0.143, θ = 0.537)* and Masked Correctness × Unmasked Correctness interactions *(F(1,25) = 7.52, p = 0.011 η_p_^2^ = 0.231, θ = 0.751)* (Figure 3). *Post hoc* analyses revealed Unmasked Correctness effects exclusively for the anterior regions *(Δ = −0.259, p = 0.043)*. This result is in line with cluster analyses that yielded stronger significances in anterior electrodes at around 350 ms. Additionally, post-hoc analyses of Masked Correctness by Unmasked Correctness interaction yielded differences for I_NA_-C_NV_-I_AV_ vs. I-C_NV_-C_AV_ *(Δ = −0.239, p = 0.033)* and I_NA_-C_NV_-I_AV_ vs. I_NA_-I_NV_-C_AV_ *(Δ = −0.316, p = 0.01)*, where I_NA_-C_NV_-I_AV_ condition presented a larger negativity in this window (see Figure 3). This I_NA_-C_NV_-I_AV_ larger negativity might explain the positive Unmasked Correctness effect reported above, since unmasked correct sentences agglutinaIC_NA_-C_NV_-C_AV_ and I_NA_-C_NV_-I_AV_ conditions, while unmasked incorrect sentences join I_NA_-I_NV_-C_A__I_nd C_NA_-I_NV_-I_AV_ conditions, and no significant differences were observed I tween C_NA_-I-C_AV_ and C_NA_-I_NV_-I_AV_ conditions (Figure 3). Finally, a significant main effect for Emotion was found *(F(2,50) = 3.92, p = 0.027, η_p_^2^ = 0.136, θ = 0.674)*, where positive and negative conditions were significantly different *(Δ = −0.261, p = 0.043)* (Figure 4). All other factors or interactions did not reach statistical significances *(all Fs < 3.1, ps > 0.09).*

#### Time Window 470–550 ms

**FIGURE 5:**
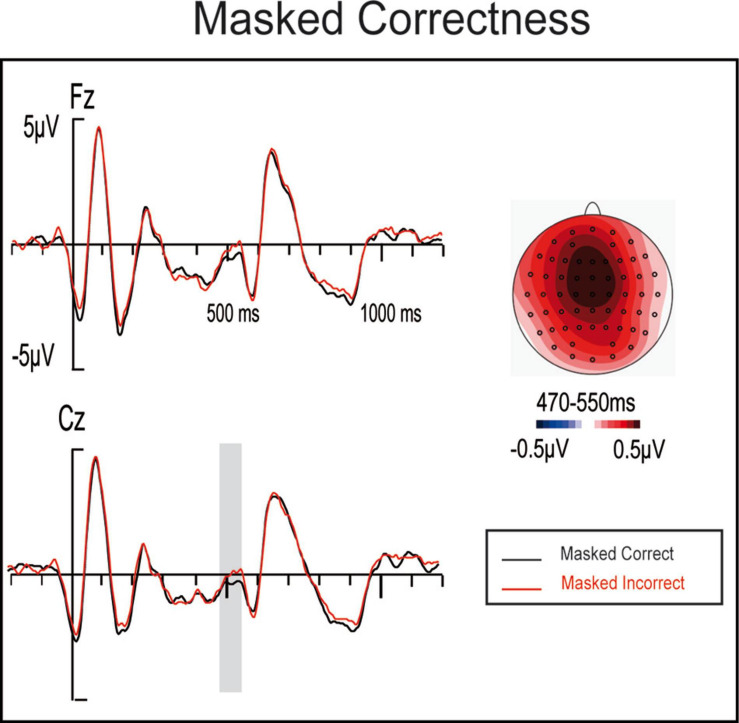
ERP waveforms and maps for masked correct sentences and masked incorrect ones.

**FIGURE 6:**
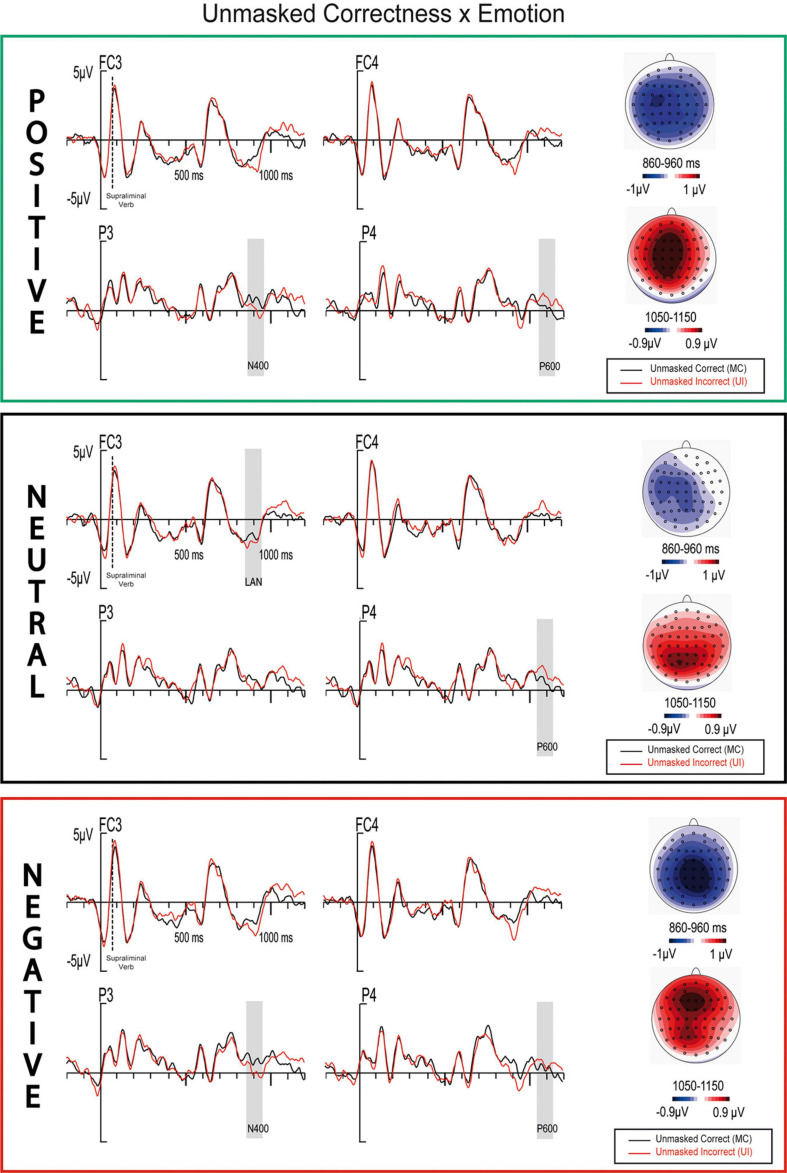
ERP waveforms and maps for correct and incorrect sentences containing either positive, negative, or neutral masked adjectives.

In this window, cluster analyses yielded a main effect of emotion (Supplementary Figure 1), while windows analyses revealed only a masked Correctness effect *(F(2,50) = 4.41, p = 0.046, η_p_^2^ = 0.15, θ = 0.524).* Furthermore, topographic and visual ERP explorations revealed a widespread positivity stronger at central electrodes resembling a weak P600 effect, when comparing correct and incorrect masked anomalies (Figure 5). Although this result should be taken with caution, both ANOVA analyses and visual inspections supported a possible P600 component to masked anomalies rather than an Emotional effect (Figures 5, 6). Further significant main effects or interactions were not observed for windows analyses *(all Fs < n 3.1, ps > 0.07).*

#### Time Window 860–960 ms

**FIGURE 7:**
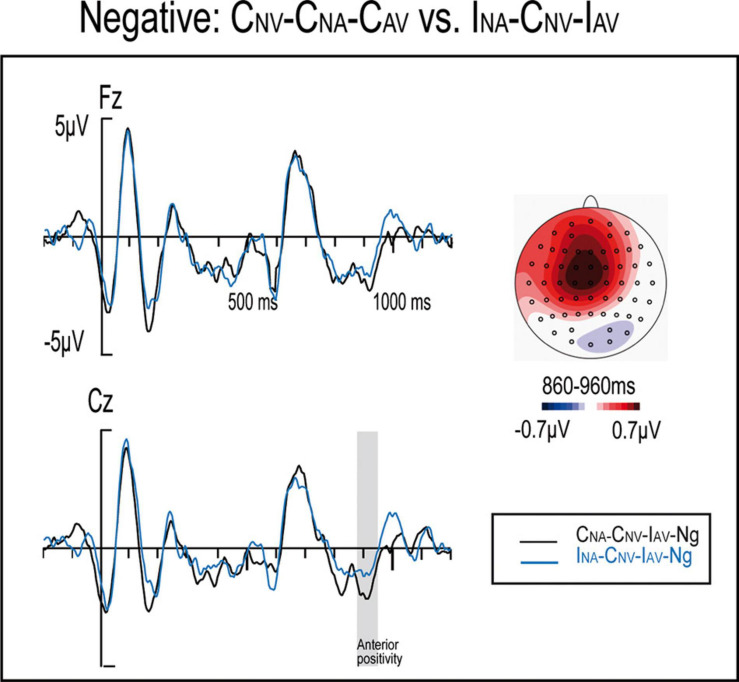
ERP waveforms and maps for Masked Correct-Unmasked Correct (C_NA_-C_NV_-C_AV_) and Masked Incorrect-Unmasked Correct (I_NA_-C_NV_-I_AV_) both negative conditions.

In this window, similarly to cluster analyses, an Unmasked Correctness significant effect was observed *(F(1,25) = 21.2, p* < 0*.001, η_p_^2^ = 0.46, θ = 0.993)*. Additionally, Unmasked Correctness × Emotion *(F(2,50) = 4.12, p = 0.024, η_p_^2^ = 0.14, θ = 0.689)* and Region × Masked Correctness × Unmasked Correctness × Emotion *(F(4,100) = 3.37, p = 0.042, η_p_^2^ = 0.13, θ = 0.612)* significant interactions also arose. *Post hoc* analyses found significant Unmasked Correctness effects (incorrect vs. correct sentences) for positive and negative adjectives (*Δ = −0.609, p = 0.001; Δ = −0.743, p* < 0*.001)*, but not for neutral ones *(Δ = −0.271, p = 0.11).* Both presented topographies similar to an N400 component maximal at parietal electrodes (Figure 6). Nonetheless, ERP waveforms and topographic maps inspections for the neutral condition suggested the presence of a weak negative anterior component to unmasked anomalies, maximal at FC5, FC3, and C5 electrodes, which topography (more left anterior than for negative and positives) resembles a weak LAN component (Figure 6). Furthermore, an Electrode (3) × Unmasked Correctness (2) × Masked correctness ANOVA in neutral adjectives disclosed significances between unmasked correct and unmasked incorrect trials *(F(4,100) = 4.44, p = 0.045, η_p_^2^ = 0.15, θ = 0.53)*. Finally, to disentangle the quadruple interaction described above, *post hoc* analyses were calculated. Within negative adjectives, there was a significant difference between can-I_NV_-I_AV_-Ng acanC_NA_-C_NV_-C_AV_-Ng conditions *(Δ = 0.329, p = 0.02)*; difference maps showed a positivity at anterior regions (Figure 7). All other factors and interactions in this window were not significant *(all Fs < 2.5, ps > 0.11).*

#### Time Window 1050–1150 ms

In line with cluster analyses, main effects for Unmasked Correctness and for the interaction Hemisphere × Unmasked Correctness were significant (*F(1,25) = 16.66, p* < 0*.001, η_p_^2^ = 0.4, θ = 0.975; F(1,25) = 5.83, p = 0.023, η_p_^2^ = 0.189, θ = 0.642).* In this case, the topography resembles a P600 component, though more central than expected (Figure 6).

## Discussion

The present study aimed to explore the possible double nature of syntactic parsing: automatic, but flexible and adaptable to context. Specifically, what is investigated is the processing of masked emotional adjectives containing morphosyntactic anomalies (number agreement) and the extent to which they affect the linguistic comprehension of ongoing unmasked sentences, which also contain number agreement anomalies in the verb. We found that no differences in behavioral data were not found, possibly due to the delay included to prevent muscular artifacts between sentence presentation and participants’ response. However, ERP modulations were observed to emotional information, both to masked and to unmasked anomalies. Furthermore, interactions between the processing of conscious and unconscious morphosyntactic anomalies as well as between unconscious emotional information and conscious morphosyntactic anomalies were detected. Bellow, findings for each time window are discussed.

### Attentional/Priming Effects

A negativity appeared in the 150–230 ms window, which was smaller for correct masked adjectives than for masked incorrect ones. This effect might probably appear because the amplitude was significantly smaller for the I_NA_-I_NV_-C_AV_ than for the C_NA_-I_NV_-I_AV_ conditions, as the masked Incorrect variable agglutinates I_NA_-I_NV_-C_AV_ and I_NA_-C_NV_-I_AV_ conditions, while the masked Correct variable agglutinates C_NA_-C_NV_-C_AV_ and C_NA_-I_NV_-I_AV_. This effect could be interpreted as a N1 component, since it appeared at around 100 ms after the unmasked verb presentation. The N1 component is said to reflect attentional and discrimination processes (Luck et al., 1996; Vogel and Luck, 2000). The N1 effects could arise because of the conflict or mismatch produced by a masked correct adjective followed by an unmasked incorrect verb, while in the masked incorrectness might prime the appearance of the unmasked incorrectness in the I_NA_-I_NV_-C_AV_ condition, and thus capturing fewer attentional resources. Furthermore, verb anomalies preceded by emotional stimuli have been reported to elicit the N1 (Verhees et al., 2015). The N100 component has also been found for German–French L2 for gender disagreements (Foucart and Frenck-Mestre, 2011). These findings suggest that the N100 is a very early and highly automatic sign of syntactic processing, more common when using pair of words or even mismatch paradigms (Hasting and Kotz, 2008; Pulvermuller et al., 2008; Foucart and Frenck-Mestre, 2011). This data might also point out that the observed N100 component might appear due to the mismatch between the masked adjective and the unmasked verb, regardless of the rest of the sentence.

The N1 effect was not initially predicted in the present study, because N1 modulations were not observed in a previous experiment wherein masked adjectives containing morphosyntactic anomalies were presented embedded in a conscious sentence (Jiménez-Ortega et al., 2014). The sentence stimuli of that experiment presented an unmasked adjective for 17 ms that could contain an anomaly, followed by a masked adjective. As such, the masked and unmasked information were somehow either contradictory or redundant from a grammatical point of view. In contrast, in the present study, the masked anomaly at the adjective could prime the coming noun–verb number disagreement. This would then result in the observed amplitude reduction for I_NA_-I_NV_-C_AV_ condition in comparison to C_NA_-I_NV_-I_AV_.

### Masked Emotional Effects

Firstly, an emotional effect was observed when comparing positive and negative adjectives at around 370 ms (Figure 4). Its timing and the topography, although more widespread and central, resemble those of an emotional EPN-like component (Herbert et al., 2008; Kissler et al., 2009; Schupp et al., 2013; Bayer and Schacht, 2014; Espuny et al., 2018a). This is supported by the stronger significances at frontal and parietal electrodes found in the cluster analyses. This topography closely aligns with that of EPN components. It should be noted that mastoids reference was used to favor morphosyntactic effects, instead of the traditional average reference used in emotional language research, which might explain why the topography did not match entirely (Rellecke et al., 2013). The EPN for words is said to reflect voluntary orientation and attention toward words with evident emotional significance at early processing stages, in which the task-relevant stimuli are selected for further, more elaborate processing (Kissler et al., 2006; Potts et al., 2008; Conrad et al., 2011). In this study, however, the EPN was triggered to masked emotional words, without awareness. In this case, voluntary orientation might not be necessary but rather a resource mobilization. Further research is needed using masked emotional words to disentangle the nature of the EPN. In any case, the presence of these modulations in response to our masked emotional stimuli guarantees the efficacy of our procedures for eliciting masked word processing and the intended emotional effects, regardless of the masked morphosyntactic processing.

### The Automatic Nature of Syntax

A main masked Correctness effect appeared at around 330–430 ms, which might support the automatic nature of syntactic. Specifically, a I_NA_-C_NV_-I_AV_ larger negativity was observed in comparison to both the C_NA_-C_NV_-C_AV_ and the I_NA_-I_NV_-C_AV_ conditions, which as detailed in the result section might explain the masked Correctness effect. The topography and timing of both negativities resemble those of the left anterior negativity, but probably arising due to different underlying mechanisms, discussed below.

The LAN observed for the I_NA_-C_NV_-I_AV_ vs. C_NA_-C_NV_-C_AV_ comparison might exclusively appear as a result of the automatic processing of masked morphosyntactic anomalies, as reported in Jiménez-Ortega et al. (2014). In their study, masked gender agreement violations triggered an early anterior negativity. This suggests that syntactic processing might occur at early stages, even under unconscious conditions. Although, as discussed above, earlier N100 modulations to syntactic anomalies have been observed in impoverished contexts (e.g., pair of words) (Foucart and Frenck-Mestre, 2011), both findings support the hypothesis that anterior negativities might reflect the most automatic and unaware part of syntactic sentence processing (Hasting and Kotz, 2008; Batterink et al., 2010). Therefore, first-pass syntactic parsing appears to be unconsciously and automatically elicited.

The comparison between the I_NA_-C_NV_-I_AV_ vs. I_NA_-I_NV_-C_AV_ conditions reveals an effect whose timing and a topography resemble those of the LAN component. It should be noted that in the I_NA_-I_NV_-C_AV_ condition, the only disagreement occurs with the noun: the masked adjective and unmasked verb agree with each other. Furthermore, according to the ERP data analyses, the I_NA_-I_NV_-C_AV_ condition may behave like the C_NA_-C_NV_-C_AV_ condition, since no significant differences were detected between them in any window. This result might indicate that, regardless of the level of awareness, grammatical information might be processed jointly. Furthermore, it might even confirm the automaticity of syntactic processing (Hasting and Kotz, 2008; Batterink et al., 2010; Jiménez-Ortega et al., 2014), as the agreement between the masked adjective and unmasked verb seems to prevail over the disagreement between the noun and the verb of the conscious sentence, at least at initial stages of syntactic processing. Similarly, Jiménez-Ortega et al. (2014) also observed a reduction of amplitude for masked incorrect and unmasked incorrect adjectives in comparison to masked incorrect and unmasked correct adjectives. Additionally, as in the N1 component, the incorrect masked adjective might prime the coming noun–verb number disagreement resulting in an amplitude reduction observed for the I_NA_-I_NV_-C_AV_ condition. Berkovitch and Dehaene (2019) found in a behavioral study that prime-target congruity in grammatical number could induce a strong priming effect under both unmasked and masked conditions, resulting in no longer significant reaction time differences between, for example, the grammatically correct “des reptiles” (“some reptiles”) and the grammatically incorrect “ils reptiles” (“they reptiles”).

A masked main effect of correctness appeared at around 490 ms according to the window analyses, while the cluster analyses detected an emotional main effect instead. These discrepancies between these analyses may be because main factors are not direct measures; rather, they are calculated by combining several experimental conditions. Visual inspections supported a masked correctness effect rather than an emotional one (Figures 5, 6) that might resemble a P600-like component, although with a more fronto-central distribution than expected. While this result should be taken with caution, a similar one was observed by Jiménez-Ortega et al. (2014), where a P600-like component was observed to masked gender anomalies. Both results might support that not all the processes reflected by the P600 would be controlled, since a weak component seems to be triggered by unaware syntactic anomalies automatically processed. As hypothesized in Jiménez-Ortega et al. (2014), the P600 component might be a general index of linguistic information integration, where not only semantics and syntax are combined but also conscious and unconscious information.

Altogether, anterior negativities and a weak P600 component appeared to unconscious syntactic anomalies, supporting the automatic nature of syntax. This is in line with previous proposals that even conceived syntax as reflex (Shtyrov et al., 2003; Hasting and Kotz, 2008; Pulvermuller et al., 2008; Batterink and Neville, 2013).

### The Flexible and Content-Dependent Nature of Syntax

A main Unmasked Correctness effect arose at around 910 ms with a widespread central negativity, which might reflect a N400 component to morphosyntactic anomalies (for similar N400 topographies and functional significance see: Molinaro et al., 2015; Fromont et al., 2020; Jiménez-Ortega et al., 2020). However, the onset of the component seems to be somehow delayed in comparison to other recent studies using similar sentences (Hinchcliffe et al., 2020; Jiménez-Ortega et al., 2020), wherein LAN/N400 components to syntactic anomalies are generally triggered at around 400 ms. It should be noted that the unmasked incorrectness occurred 390 ms just after the masked adjective onset; therefore, an unmasked LAN/N400 component should have arisen at around 790 ms instead of at 910 ms. Most likely, both the delay and the N400-like distribution appeared as a result of the processing of the masked adjective. Previous studies that presented masked adjectives did not observe such a long delay (Jiménez-Ortega et al., 2014, 2017). In these studies, there were masked adjectives and unmasked adjectives presented almost simultaneously (17 ms delay), which is a correct but uncommon grammatical structure. In contrast, in the present study, there was just one masked adjective followed by the verb of the unmasked sentence. Therefore, the masked adjective might provide novel information for sentence comprehension, in contrast with previous experiments, thus slightly delaying the component onset.

An unmasked correctness effect was observed for positive and negative masked adjectives resembling an N400 component, while a weak LAN was observed for the neutral condition. Therefore, the increased amplitude together with its N400-like distribution to Unmasked Correctness might appear as a result of the adjectives emotional information, thus supporting the flexible- and context-dependent nature of syntax. The N400-like distribution might reflect a switch in the processing strategy, from an algorithmic to a heuristic one, proper of transient emotional states (e.g., Isen and Means, 1983; Blanchette and Richards, 2010; Jiménez-Ortega et al., 2020). Similarly, both masked and unmasked positive adjectives have elicited an N400 instead of a LAN component in response to morphosyntactic violations in previous studies (Martin-Loeches et al., 2012; Jiménez-Ortega et al., 2017). These studies support the hypothesis that emotional unconscious information might induce heuristic processing strategies (Holt et al., 2009), since the N400 component may reflect a more heuristic-like processing, while the LAN component might reflect purer algorithmic and rule-based strategies (Martin-Loeches et al., 2009; Jiménez-Ortega et al., 2020).

Regardless of the level of awareness, the direction of the emotional ERP modulations on syntax is not well understood yet. In line with the present results, previous studies also found that positive and negative emotional stimuli behaved similarly at early stages among them, and often found differences when compared to neutral information (Jiménez-Ortega et al., 2012; Espuny et al., 2018a; Padron et al., 2020). In contrast, other studies found differences in the early components to syntactic anomalies between positive and negative emotional information (Martin-Loeches et al., 2012; Jiménez-Ortega et al., 2017; Espuny et al., 2018b). Particularly, in Jiménez-Ortega et al. (2017), emotional masked adjectives, also embedded within a sentence, preceded an unmasked neutral adjective containing a gender or a number anomaly. As in the current experiment, the neutral condition elicited a left anterior negativity followed by a P600 component. However, there was no anterior negativity in the negative condition, and an early P600 onset was observed instead, while the positive condition elicited an N400 component. In contrast with the present study, where levels of arousal were matched, the positive adjectives employed in Jiménez-Ortega et al. (2017) presented lower levels of arousal (*M* = 4.9, *SD* = 0.8) than the negative ones (*M* = 6.3, *SD* = 0.6). Furthermore, in the present study, the unmasked incongruences were presented at the verb instead of the adjective, and sentences were larger. Most likely, considering the importance of both valence and arousal during emotional words processing (Citron et al., 2013), the differences between the two studies could be explained by the material disparities in arousal levels. Similar to our results, a recently conducted study presented emotional adjectives consciously (Padron et al., 2020). Although a neutral condition was not included, the early components—a LAN in this case, observed for moderately arousing pleasant and unpleasant adjectives containing syntactic anomalies—did not differ. As discussed above, our results support that, regardless of the exact contribution of valence and arousal, emotional information when compared to neutral one seems to modulate the amplitude and topography of early syntactic components, supporting the flexible nature of syntax.

An anterior positivity was also observed at the 860–960 ms window when comparing the I_NA_-C_NV_-I_AV_-Ng and the C_NA_-C_NV_-C_AV_-Ng conditions, i.e., within unmasked correct sentences, there were significant differences when comparing masked incorrect and masked correct negative adjectives. Although this difference appeared as a result of a quadruple interaction, which are commonly difficult to disentangle and caution should be taken, it might be due to a correct but unexpected unmasked correctness. For example, DeLong et al. (2014) observed a positivity at anterior regions to unexpected but correct words. Similarly, the masked anomaly might have primed/preactivated the error detection system, which might be prepared to find an unmasked anomaly. Therefore, the subsequent unmasked correct verb might appear as correct but unexpected, resulting in the observed anterior positivity. This effect might arise exclusively for the negative condition, since negative emotions seem to particularly boost analytic behavior strategies (Park and Banaji, 2000) that might help to error detection. Alternatively, negative words might predict better the appearance of an incorrectness via an implicit association between incorrectness and unpleasantness. Nonetheless, although further studies should be carried out to confirm this finding and its implications, this result might also support the flexible and context-dependent nature of syntactic processing.

Finally, a main effect of unmasked Correctness resembling a P600 component arose at around 1100 ms. This component was more delayed, central, and weaker than expected to syntactic anomalies using similar sentences (Jiménez-Ortega et al., 2017, 2020; Hinchcliffe et al., 2020). Yet, a larger early syntactic component and a reduced P600 have been previously observed for good vs. poor comprehenders (Coulson and Kutas, 2001). Furthermore, similarly to the present study, the same pattern appears when emotional words precede sentences containing a morphosyntatic violation, compared to neutral information (Espuny et al., 2018b). The increased early component/reduced P600 pattern is therefore generally interpreted as a result of a more efficient syntactic information processing (Tanner and Van Hell, 2014). In the present study, the preceding masked emotional information (two-thirds of the masked adjectives) might facilitate the processing, as occurred in Espuny et al. (2018b), given the observed larger N400 to unmasked anomalies for negative and positive conditions. Additionally, according to Tanner and Van Hell (2014) results, the LAN and the N400 might be functionally equivalent, and biphasic ERP waveforms (LAN/P600) do not always reflect separable processing stages within individuals. Consequently, it is also possible that the delayed N400 to conscious morphosyntactic anomalies might allow an almost complete detection and resolution of the syntactic anomaly at this point, thus resulting in a reduction of the later P600 component.

Altogether, both the components’ delay and the N400-like distributions triggered by the noun–verb conscious syntactic anomalies seem to appear as a result of the modulations produced by the presentation of preceding masked emotional adjectives. These findings support the flexible nature but context dependence (even unconscious) of syntax processing. This is in line with previous findings where linguistic emotional information (content dependent) and extralinguistic information (context-dependent) modulated syntax processing, regardless of awareness (Vissers et al., 2010; Martin-Loeches et al., 2012; Hinojosa et al., 2014; Verhees et al., 2015; Jiménez-Ortega et al., 2017, 2020; Hinchcliffe et al., 2020).

A main limitation of the present study is the use of a limited sample size for the number of factors analyzed. Limited sample size is a common problem when using neuroimaging and EEG techniques. This problem can be minimized by using homogeneous samples, including more than 20 participants, intrasubject designs, and tasks with accuracy rates larger than 80% (Picton et al., 2000; Yarkoni, 2010). Our study fulfills all these requirements. Additionally, we have provided both effect size and power in the results to give the reader an objective measure of the reliability of the results. Further, our results were confirmed by the use of two different statistical approaches (ANOVAs and cluster analysis). Cluster analysis, due to its characteristics, does not include topographic factors, thus compensating somehow the power limitation of the ANOVA analyses. Both cluster and ANOVA analyses matched, both being significant in the same windows and factors, with the two exceptions already commented and for which cautions have been raised.

## Conclusion

Both conscious and unconscious anomalies triggered early syntactic components and interacted between them, thus supporting the automatic nature of first-pass syntactic parsing. Furthermore, the unconscious emotional content also modulated the early syntactic component to conscious anomalies, supporting the flexible, permeable, and content-dependent nature of the syntactic processing. However, further research is needed to better disentagle the direccion and implications of the emotional modulations. Nonetheless, this double nature of syntax is in line with theories of automaticity suggesting that even unconscious/automatic processing is flexible, adaptable, and context-dependent (for reviews, see: Kiefer, 2012; Ansorge et al., 2014). Finally, although further research might be needed, the interaction observed between masked Correctness and unmasked Correctness might point out that both conscious and unconscious information is combined by the automatic syntactic system.

## Data Availability Statement

The raw data supporting the conclusions of this article will be made available by the authors, without undue reservation.

## Ethics Statement

The studies involving human participants were reviewed and approved by the Comisión Deontológica de la Facultad de Psicología, Universidad Complutense de Madrid. Ref. 2016/17-021. The patients/participants provided their written informed consent to participate in this study.

## Author Contributions

LJ-O and MM-L developed the study concept and designed the study. EB, LJ-O, SF, PC, FM, DH-G, JS-G, and MM-L collected the data, interpreted the data, and discussed the results. EB and LJ-O prepared and analyzed the data and wrote the manuscript. All authors revised the manuscript.

## Conflict of Interest

The authors declare that the research was conducted in the absence of any commercial or financial relationships that could be construed as a potential conflict of interest.

## Supplementary Material

Supplementary Figure 1 Significant outputs of Cluster calculation for 0–600 ms window, including factors Masked Correctness (2) × Unmasked Correctness (2) × Emotion (3) by using the Factorial Mass Univariate Toolbox (Fields and Kuperberg, 2019). From top to down: Unmasked Correctness, Emotion, and Masked Correctness × Unmasked Correctness effects. Right scale represents observed significant *F*-values (*p* < −0.5) at each electrode and time window for each factor and interaction.

Supplementary Figure 2 Significant outputs of Cluster calculation for 600–1200 ms window, including factors Masked Correctness (2) × Unmasked Correctness (2) × Emotion (3) by using the Factorial Mass Univariate Toolbox (Fields and Kuperberg, 2019). Right scale represents observed significant *F*-values (*p* < −0.5) at each electrode and time window for each factor and interaction.
