# Supplementary figures and images for: The Automatic but Flexible and Content-Dependent Nature of Syntax
Source: Front Hum Neurosci. 2021 Jun 11;15:651158. doi: 10.3389/fnhum.2021.651158 (PMC8226263; doi:10.3389/fnhum.2021.651158)

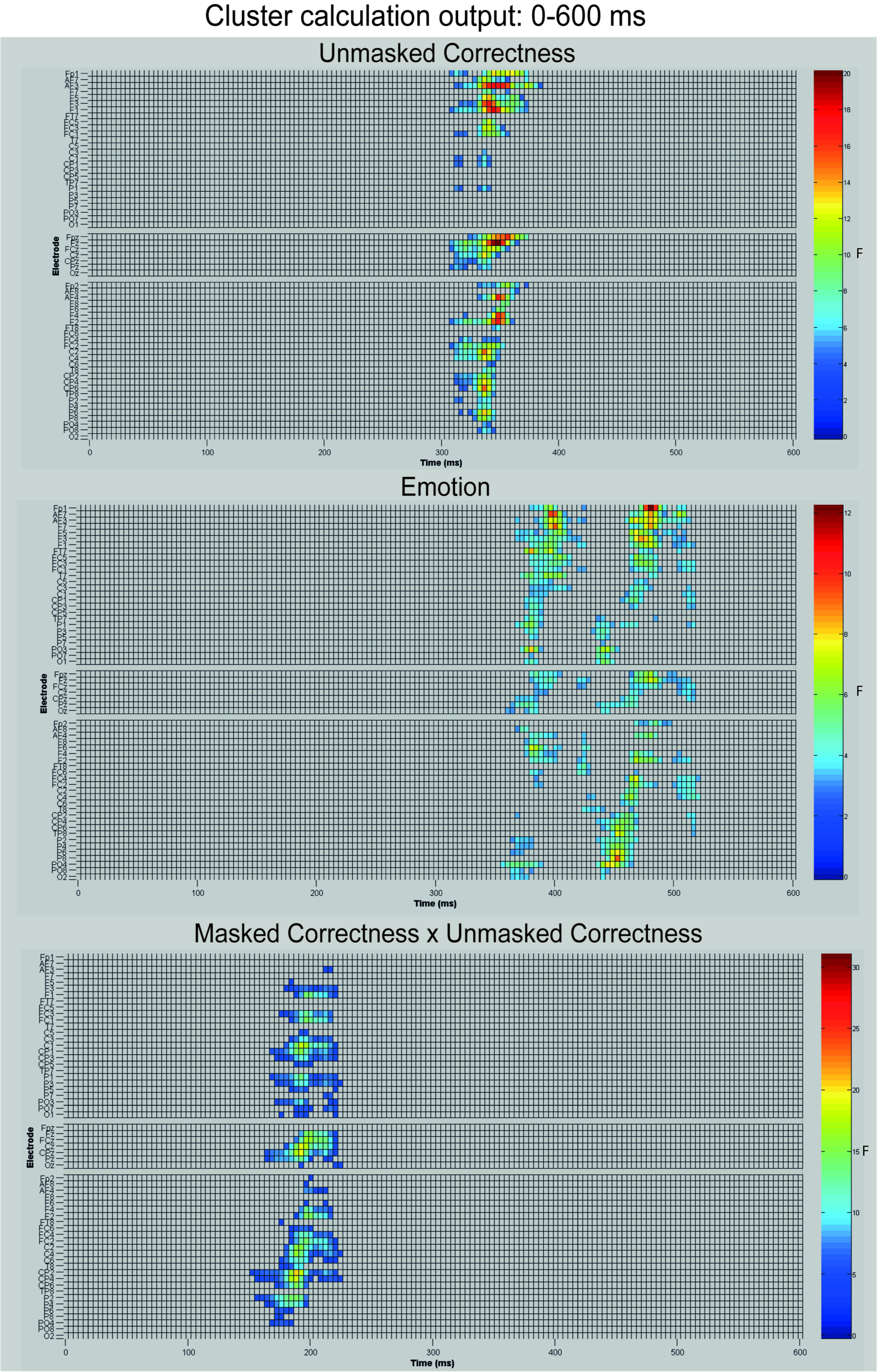

Supplement: Supplementary Figure 1 — Significant outputs of Cluster calculation for 0–600 ms window, including factors Masked Correctness (2) × Unmasked Correctness (2) × Emotion (3) by using the Factorial Mass Univariate Toolbox (Fields and Kuperberg, 2019). From top to down: Unmasked Correctness, Emotion, and Masked Correctness × Unmasked Correctness effects. Right scale represents observed significant F-values (p < −0.5) at each electrode and time window for each factor and interaction. [file Image_1.tif]

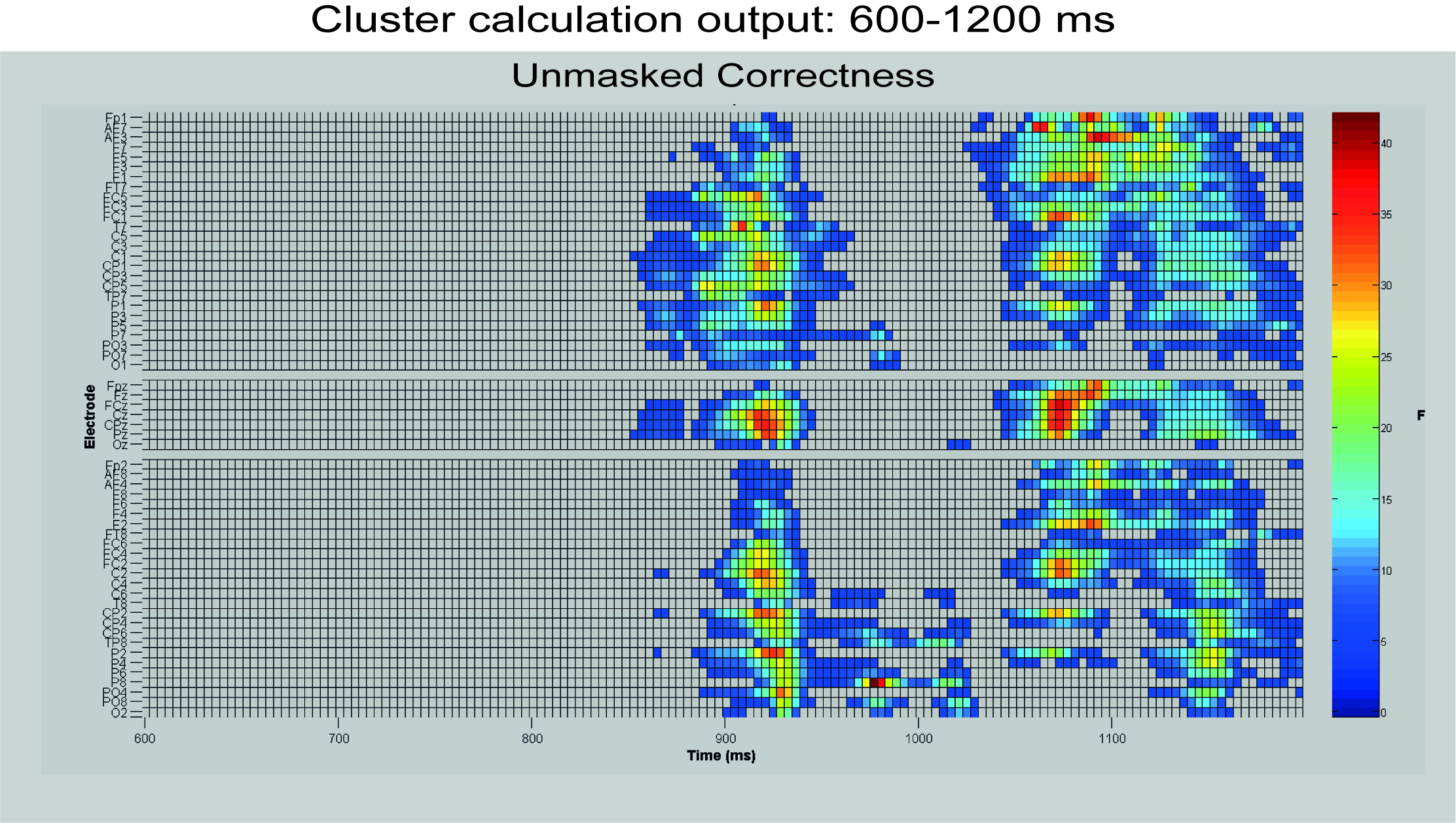

Supplement: Supplementary Figure 2 — Significant outputs of Cluster calculation for 600–1200 ms window, including factors Masked Correctness (2) × Unmasked Correctness (2) × Emotion (3) by using the Factorial Mass Univariate Toolbox (Fields and Kuperberg, 2019). Right scale represents observed significant F-values (p < −0.5) at each electrode and time window for each factor and interaction. [file Image_2.tif]
